# Overexpression of Akt1 Enhances Adipogenesis and Leads to Lipoma Formation in Zebrafish

**DOI:** 10.1371/journal.pone.0036474

**Published:** 2012-05-18

**Authors:** Che-Yu Chu, Chi-Fang Chen, R. Samuel Rajendran, Chia-Ning Shen, Te-Hao Chen, Chueh-Chuan Yen, Chih-Kuang Chuang, Dar-Shong Lin, Chung-Der Hsiao

**Affiliations:** 1 Department of Bioscience Technology, Chung Yuan Christian University, Chung-Li, Taiwan; 2 Genome Research Center, Academia Sinica, NanKang, Taiwan; 3 National Museum of Marine Biology and Aquarium, Pingtung, Taiwan; 4 Division of Hematology & Oncology, Department of Medicine, Taipei Veterans General Hospital, Taipei, Taiwan; 5 National Yang-Ming University School of Medicine, Taipei, Taiwan; 6 Division of Genetics and Metabolism, Department of Medical Research, Mackay Memorial Hospital, Taipei, Taiwan; 7 Department of Pediatrics, Mackay Memorial Hospital, Taipei, Taiwan; 8 Department of Medical Research, Mackay Memorial Hospital, Taipei, Taiwan; 9 Mackay Medicine, Nursing and Management College, Taipei, Taiwan; 10 Department of Chemical Engineering and Biotechnology, National Taipei University of Technology, Taipei, Taiwan; 11 Center for Nanotechnology, Chung Yuan Christian University, Chung-Li, Taiwan; Children’s Hospital & Harvard Medical School, United States of America

## Abstract

**Background:**

Obesity is a complex, multifactorial disorder influenced by the interaction of genetic, epigenetic, and environmental factors. Obesity increases the risk of contracting many chronic diseases or metabolic syndrome. Researchers have established several mammalian models of obesity to study its underlying mechanism. However, a lower vertebrate model for conveniently performing drug screening against obesity remains elusive. The specific aim of this study was to create a zebrafish obesity model by over expressing the insulin signaling hub of the *Akt1* gene.

**Methodology/Principal Findings:**

*Skin oncogenic transformation screening shows that a stable zebrafish transgenic of Tg(krt4Hsa.myrAkt1*)^cy18^ displays severely obese phenotypes at the adult stage. In Tg(*krt4:Hsa.myrAkt1*)^cy18^, the expression of exogenous human constitutively active Akt1 (myrAkt1) can activate endogenous downstream targets of mTOR, GSK-3α/β, and 70S6K. During the embryonic to larval transitory phase, the specific over expression of myrAkt1 in skin can promote hypertrophic and hyperplastic growth. From 21 hour post-fertilization (hpf) onwards, myrAkt1 transgene was ectopically expressed in several mesenchymal derived tissues. This may be the result of the integration position effect. Tg(*krt4:Hsa.myrAkt1*)^cy18^ caused a rapid increase of body weight, hyperplastic growth of adipocytes, abnormal accumulation of fat tissues, and blood glucose intolerance at the adult stage. Real-time RT-PCR analysis showed the majority of key genes on regulating adipogenesis, adipocytokine, and inflammation are highly upregulated in Tg(*krt4:Hsa.myrAkt1*)^cy18^. In contrast, the myogenesis- and skeletogenesis-related gene transcripts are significantly downregulated in Tg(*krt4:Hsa.myrAkt1*)^cy18^, suggesting that excess adipocyte differentiation occurs at the expense of other mesenchymal derived tissues.

**Conclusion/Significance:**

Collectively, the findings of this study provide direct evidence that Akt1 signaling plays an important role in balancing normal levels of fat tissue in vivo. The obese zebrafish examined in this study could be a new powerful model to screen novel drugs for the treatment of human obesity.

## Introduction

Obesity has become a worldwide health problem in recent decades. According to the International Obesity Task Force (IOTF), the worldwide overweight population now exceeds 1.7 billion. The potential risks of obesity or metabolic syndrome will affect human health and life quality. In addition, chronic diseases like diabetes, hypertension, hyperlipidemia, cardiovascular disease, and cancer are directly associated with obesity [1], [2], [3], [4], [5]. However, the mechanism for inducing obesity is unclear. Evidence collected from recent studies indicates that obesity involves complex physiological disorders and is influenced by the interaction of genetic, epigenetic, and environmental factors [6], [7], [8]. To simplify the complexity, current methods of probing adipogenesis, obesity-related chronic diseases and metabolic syndrome largely rely on conventional in vitro cell culture and in vivo animal models. Cell culture can manipulate culture conditions to evaluate the biological effects of exogenous drug or hormone treatment on adipocyte differentiation. For example, 3T3-L1 fibroblast cells [9] and embryonic stem (ES) cells [10], [11] can be efficiently induced to adipocyte cells when retinoic acid (RA) and adipogenic hormones are administrated in vitro. However, since obesity is the dysregulated outcome of multiple physiological processes on several target organs, it is insufficient to explore the physiological or cellular mechanisms of human obesity using an in vitro approach. Therefore, researchers have successfully developed several animal models in nematodes [12], [13], flies [14], [15] and rodents [16], [17] to probe adipogenesis and the disease mechanism of human obesity in vivo.

The conventional murine model provides a gold standard for human obesity research. For example, mice deficient in *leptin* (*ob/ob*) [18], [19], [20], [21], *leptin receptor* (*db/db*) [22], [23] or *Ankrd26* [16] genes spontaneously display an obese phenotype because their appetite is out of control, leading to extra food intake. It is also possible to explore novel and evolutionary conserved genes on controlling lipid metabolic pathway and adipocyte development by performing large-scale forward and reverse genetic screening in worms and flies [24], . However, because of the distinct anatomy of higher vertebrates, it is not possible to measure some physiological parameters, such as blood glucose levels and insulin resistance, in worms and flies. Therefore, it is necessary to develop a lower vertebrate obesity model to overcome this bottleneck and accelerate research.

The zebrafish model has recently emerged as a new and attractive animal model for human disease and obesity [26], [27], [28]. Several lines of evidence support the idea that zebrafish allow the study of adpiogenesis and modeling of human obesity. First, the nile red staining method of detecting lipids in living fish has been established and can be used to follow the adipogenesis process in real time [29]. Second, the cellular anatomy of zebrafish adipocytes is similar to mammalian white adipocytes, and most of the marker genes in adiopogenesis or lipogenesis pathway have been characterized [29]. Third, the method of measuring blood glucose in zebrafish has been established [30], [31], making it possible to use zebrafish as a new powerful animal model for probing human obesity. Fourth, transcriptomic studies indicate a similarity in the gene expressional profiling of diet-induced obesity between fish and mammals [28]. Finally, Song and Cone demonstrated that the overexpression of endogenous melanocortin antagonist agouti-related protein (AgRP) in transgenic zebrafish can enhance appetite and induce obese phenotype in live zebrafish [32]. These results demonstrate that the key components of the adipostat are conserved between fish and mammals, highlighting fish as an alternative and inexpensive animal model for human obesity.

Akt (also known as protein kinase B, PKB) is a serine/threonine protein kinase that regulates cell survival, cell growth, cell cycle, cell proliferation, cell metabolism, cell migration/invasion, angiogenesis, and functions an essential hub gene to crosstalk with numerous signaling pathways [33], [34], [35]. The phosphatidylinositol 3-kinase (PI3-K)/Akt signaling pathway also plays a key role in regulating adipocyte differentiation and lipogenesis in vitro [36], [37], [38]. The enforced expression of the constitutively active form of Akt1 (myrAkt1) in 3T3-L1 pre-adipose cells can promote spontaneous adipose differentiation [39]. In contrast, mice lacking Akt1 has reduced body size and impeded subcutaneous adipogenesis [40], [41]. These results underline the pivotal role of Akt1 in adipogenesis regulation and organism development. Although the Akt1 functions on adipogenesis and fat homeostasis have been extensively studied, few studies have investigated whether they also play a role in lower vertebrate of fish. In our lab, we have performed a large-scale screen to evaluate the oncogenic transformation potential of human genes in zebrafish. To our surprise, we identified that a transgenic line of Tg(*krt4:Hsa.myrAkt1*)^cy18^ displays an obese phenotype. This study describes the phenotype of Tg(*krt4:Hsa.myrAkt1*)^cy18^ on morphological and molecular levels. Results reveal that the Akt1 pathway plays a key role in adipogenesis in vivo. Thus, this study provides a new lower vertebrate model to study human obesity.

## Results

### Generation of Tg(*krt4:Hsa.myrAkt1*)^cy18^ Zebrafish Line

**Figure 1:**
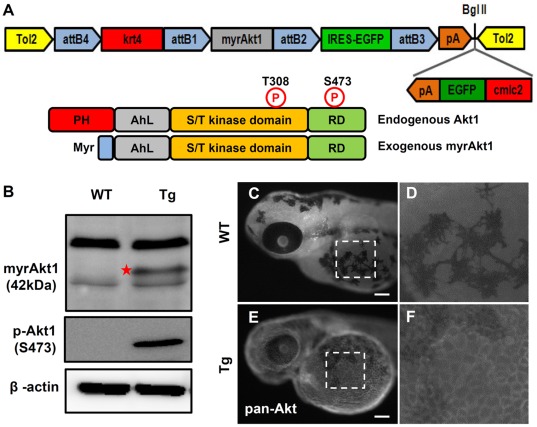
Generation of Tg*(krt4:Hsa.myrAkt1*)^cy18^. (A) The schematic diagram in the upper panel shows the configuration of the pDestTol2CG2-krt4-myrAkt1-IRES-EGFP-pA plasmid used to generate Tg*(krt4:Hsa.myrAkt1*)^cy18^. The parental vector of pDestTol2CG2, which contains *cmlc2-*EGFP-pA mini-gene, helps transgenic progeny to express green fluorescent protein in the heart. The lower panel illustrates the domain structure of the wild-type and constitutively active form of human Akt1. (B) Western blot analysis of protein lysate extracted from adult tail fins shows that the exogenous human myrAkt1 (indicated by star) is only detectable in Tg*(krt4:Hsa.myrAkt1*)^cy18^. *β*-actin served as a loading control. Whole-mount immunostaining of wild-type (C) or Tg*(krt4:Hsa.myrAkt1*)^cy18^(E) embryos aged 3 days post-fertilization using pan-Akt antibody. The yolk sac region highlighted by a dotted line in C and E is magnified in E and F, respectively. PH, Pleckstrin homology; RD, regulation domain; Myr, myristylation signal; WT, wild type; Tg, Tg(*krt4:Hsa.myrAkt1*)^cy18^. Scale bar  = 100 µm.

We performed a large-scale screen to identify the oncogenic potential of human genes in zebrafish skin. Human genes were placed under the control of zebrafish skin-specific keratin4 (*krt4*) promoter and injected expressional vectors into one-cell stage zebrafish embryos. The injected embryos were raised to adult and the stable transgenic fish were screened by outcrossing with wild-types. To facilitate fluorescence-based phenotypic screening and enhance the germ-line transmission rate, we used Tol2Kit vector [42] that contains the cmcl2-EGFP-pA mini-cassette at the 3’end and the Tol2 transposable elements flanking the whole transgene cassette at both ends (Fig. 1A). The zebrafish endogenous 2.2 kb *krt4* promoter is able to drive transgene expression in the superficial skin layer of zebrafish [43]. The myrAkt1 is depleted of the N-terminal PH domain and fused with the myristylation signal, bypassing the need for activation of phosphoinositides 3,4,5-trisphosphate (PIP3) and PIP2 generated by PI3K (Fig. 1A). Therefore, the myrAkt1 cannot be inhibited by PTEN, a tumor suppressor that acts as a negative regulator of the PI3K pathway, and will constitutively activate the Akt1 downstream signals. The p3E-IRES-EGFP-pA encodes a cytoplasmic-targeted EGFP, followed by a SV40 late polyA signal sequence [42], and is a reporter for monitoring the myrAkt1 expression in epidermis.

To generate the transgenic zebrafish, the expressional construct was co-injected with in vitro transcribed Tol2 transposase mRNA into the one-cell stage of wild type embryos. The injected embryos were examined for the expected mosaic expression of green fluorescence protein in the heart at 28–35 hour post-fertilization (hpf). With the help of the Tol2 transposon system, we identified 15 independent lines out of 31 putative founders (germ-line transmission rate  = 48%). However, IRES did not activate very well because many transgenic lines with the green heart phenotype displayed very weak EGFP expression in their skin, or none at all. Therefore, we used the cmlc2-EGFP-pA mini-cassette to screen putative founders. Data from genotyping results confirmed the fluorescent heart is a reliable marker of stable transgenic lines because embryos with a green heart are 100% positive for krt4-myrAkt1transgene (data not shown). Next, we performed Western blot and immunostaining to determine whether the exogenous human *myrAkt1* gene is overexpressed in a skin-specific manner. Protein lysates extracted from the pooled tail fins were analyzed by SDS-PAGE and immunoblotted by total Akt1/2/3 or p-Akt1 (S473) antibodies. Results show the Akt1/2/3 antibody can recognize endogenous Akt proteins in both wild type (WT) and Tg(*krt4:Hsa.myrAkt1*)^cy18^. However, only transgenics were positive for exogenous myrAkt1 proteins (42 kDa), which showed smaller size than the endogenous one (Fig. 1B). In addition, transgenic but not WT displayed overphosphorylation at position S473 when probed with p-Akt1 antibody (Fig. 1B). Whole-mount immunostaining also confirmed the expression of myrAkt1 (membrane-bound signals) in Tg(*krt4:Hsa.myrAkt1*)^cy18^ (Figs. 1E and 1F) but absent in WT skin (Figs. 1C and 1D). Collectively, these results indicate the generation of Tg(*krt4:Hsa.myrAkt1*)^cy18^ to target human *myrAkt1* expression in the epidermal layer.

### Overexpression of *myrAkt1* is Sufficient to Induce the Hypertrophic and Hyperplastic Growth of Skin in Zebrafish Larvae

**Figure 2:**
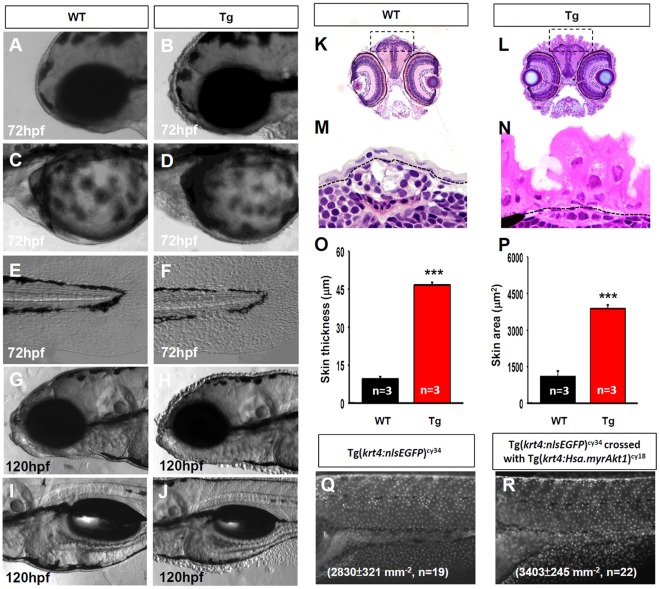
Tg*(krt4:Hsa.myrAkt1*)^cy18^ fish display skin hypertrophy and hyperplasia at the embryonic stage. Microscopic pictures of skin epidermis near the head regions (A, B), pericardial cavity and yolk sac (C, D), and tail fin (E, F) of either WT (A, C, E) or Tg*(krt4:Hsa.myrAkt1*)^cy18^ (B, D, F) at 72 hpf. At 120 hpf, the protruding appearance of skin covering the head (H) and yolk sac (J) was more pronounced in Tg than their wild-type siblings (G, I). Skin histology of WT (K, M) and Tg(L, N) embryos at 120 hpf. Cross sections through the eye position are stained with hematoxylin and eosin. The areas highlighted by a dotted line in K and L are magnified in M and N, respectively. Quantitative comparison of skin thickness (O) and area (P) between WT (black bar) and Tg (red bar). (Q, R) Quantitative comparison of skin density between Tg*(krt4:nlsEGFP*)^cy34^ and double transgenics of Tg*(krt4:nlsEGFP)* ^cy34^ *;*Tg*(krt4:Hsa.myrAkt1*)^cy18^ at 120 hpf. ***p<0.001. Data were analyzed by Student’s t-test and are shown as mean ±SEM. hpf, hour post-fertilization; WT, wild type; Tg, Tg(*krt4:Hsa.myrAkt1*)^cy18^.

The overexpression of *Akt* in a murine model can induce skin hyperplasia and promote cancer malignancy [44], [45]. This observation suggests that the overexpression of *myrAkt1* can induce cancer formation in zebrafish skin. Tg(*krt4:Hsa.myrAkt1*)^cy18^ larvae exhibited a rough organized skin phenotype from approximately 60 hpf onwards (data not shown). By 72 hpf, the scaly skin phenotype was more pronounced on the head (Fig. 2B), pericardial cavity (Fig. 2D), yolk sac (Fig. 2D), and tail fin (Fig. 2F) surface in Tg(*krt4:Hsa.myrAkt1*)^cy18^. By 120 hpf, the skin of Tg(*krt4:Hsa.myrAkt1*)^cy18^ larvae was more bulged and wrinkled than 72 hpf. This striking skin phenotype was clearly apparent even under a low power magnification (Figs. 2H and 2J). Whether this scaly skin phenotype in *myrAkt1*-overexpressing larvae was caused by skin hypertrophy or hyperplasia is an interesting topic. We answered this question by performing skin section and skin cell counting on embryos aged at 120 hpf. Compared to WT (Figs. 2K and 2M), Tg(*krt4:Hsa.myrAkt1*)^cy18^ showed a remarkable increase of skin thickness and area (Figs. 2L and 2N). Statistic measurement revealed the skin thickness (46.67±1.01 vs. 9.57±0.85 µm, n = 3, *p*<0.01, Fig. 2O) and area (3880.40±150.06 vs. 1090.80±231.35 µm^2^, n = 3, *p*<0.01, Fig. 2P) in Tg(*krt4:Hsa.myrAkt1*)^cy18^ was approximately 4- to 5-fold higher than WT siblings. In addition, plastic sections measuring 2 µm thick showed that the epidermal nuclei of EVL in Tg(*krt4:Hsa.myrAkt1*)^cy18^ were significantly larger than WT. This result strongly suggests the scaly skin phenotype in Tg(*krt4:Hsa.myrAkt1*)^cy18^ is primarily caused by skin hypertrophy. To clarify this observation, we compared the skin cell density between WT and Tg(*krt4:Hsa.myrAkt1*)^cy18^. To achieve double transgenic specimens, we crossed Tg(*krt4:Hsa.myrAkt1*)^cy18^ with Tg(*krt4:nlsEGFP*)^cy34^ line, carriers which express green fluorescence in the EVL nucleus. With the aid of the nucleus-bound nlsEGFP, it is also possible to calculate the skin cell number and density at single cell resolution in a live animal. Statistical comparison revealed a significant increase in skin cell density in Tg(*krt4:Hsa.myrAkt1*)^cy18^; Tg(*krt4:nlsEGFP*)^cy34^ (3403±245 cells/mm^2^, n = 22) (Fig. 2R) compared to Tg(*krt4:nlsEGFP*)^cy34^ (2830±321 cells/mm^2^, n = 19) (Fig. 2Q). These results indicate that overexpression of human *myrAkt1* in zebrafish epidermis can induce hypertrophic and hyperplastic growth.

### Exogenous Human *myrAkt1* Can Activate Endogenous Akt Downstream Genes in Tg(*krt4:Hsa.myrAkt1*)^cy18^

**Figure 3:**
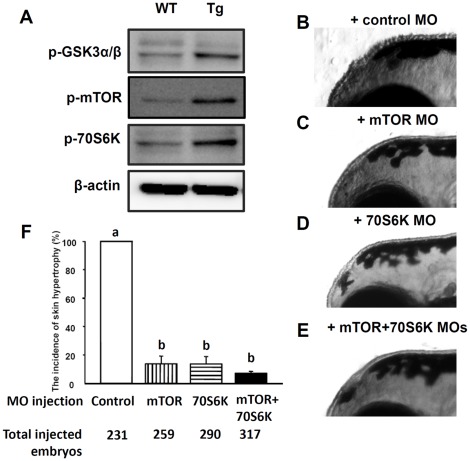
The Akt downstream genes are activated in Tg*(krt4:Hsa.myrAkt1)* ^cy18^. (A) Western blot analysis of protein lysate extracted from adult tail fins showing that many downstream targets of Akt1 are over-phosphorylated in Tg. *β*-actin served as a loading control. (B-E) MO knock-down experiment to evaluate whether the skin hypertrophy phenotype can be rescued when Akt activity ceases. Fertilized eggs collected from Tg outcross were injected with either control MO (B), mTOR MO (C), 70S6K MO (D), or mTOR+70S6K MOs (E). The skin hypertrophic appearance was scored at 120 hpf and compared quantitatively (F). MO, morpholino; WT, wild type; Tg, Tg(*krt4:Hsa.myrAkt1*)^cy18^; Different letters above the error bars indicate significant differences which tested by one-way ANOVA with Tukey’s pair-wise comparison method. hpf, hour post-fertilization.

Akt is the intermediate hub on the growth factor and insulin pathway, and plays an essential role in controlling processes such as cell size, volume, and survival by activating its downstream targets through phosphorylation [46], [47]. The observation of hypertrophic and hyperplastic transformation in Tg(*krt4:Hsa.myrAkt1*)^cy18^ larvae skin is intriguing, and leads us to ask whether this striking phenotype transformation is caused by exogenous human *myrAkt1* activity. Following this rationale, it should be able to detect the over-phosphorylation of endogenous Akt downstream targets and restore the skin phenotype when Akt downstream signaling is blocked. To validate this hypothesis, we first checked the phosphorylation status of three well known Akt downstream targets of glycogen synthase kinase 3 alpha/beta (GSK3α/β), mammalian target of rapamycin (mTOR) and 70-kDa S6 protein kinase (70S6K) using phospho-specific antibodies against each proteins. Western blot supports this speculation because the immunoreactive signals for either p-GSK3α/β, p-mTOR or p-70S6K are much stronger in Tg(*krt4:Hsa.myrAkt1*)^cy18^ than those in WT (Fig. 3A). Next, we knocked down the gene expression of Akt downstream targets of mTOR and 70S6K through gene-specific morpholino injection. Compared to the uninjected transgenics (Fig. 3B, incidence rate = 100%), the skin phenotype can be greatly attenuated in either mTOR morphants (Fig. 3C and 3F, with an incidence rate down to 17%), 70S6K morphants (Figs. 3D and 3F, with an incidence rate down to 14%) or mTOR+70S6K double morphants (Figs. 3E and 3F, with an incidence rate down to 9%). These results clearly demonstrate that the hypertrophic and hyperplastic skin growth in Tg(*krt4:Hsa.myrAkt1*)^cy18^ is caused by the upregulation of the Akt/mTOR/70S6K signaling pathway.

### Tg(*krt4:Hsa.myrAkt1*)^cy18^ Display Obese Phenotype at Adult Stage

**Figure 4:**
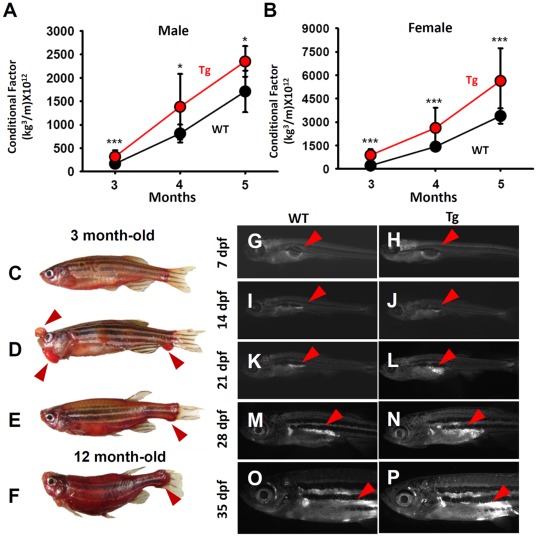
Tg*(krt4:Hsa.myrAkt1)* ^cy18^ fish display obese phenotype at the adult stage. Comparison of the conditional factor between WT (black) and Tg (red) for either male (A) or female (B) fish aged from 3- to 5-month old. Error bars labeled with stars indicate significant differences as tested by Student’s t-test. Oil Red O stains of fish from either wild-type (C), F0 founder carrying *krt4:myrAkt1*transgene (D), F1 Tg aged at 3 mpf (E), and F1 Tg aged at 12 mpf (F). Nile red vital staining reveals the lipid accumulation in WT and Tg aged at 7 dpf (G and H), 14 dpf (I and J), 21 dpf (K and L), 28 dpf (M and N) and 35 dpf (O and P). Arrows indicate nile red-positive lipids.WT, wild type; Tg, Tg(*krt4:Hsa.myrAkt1*)^cy18^; dpf, day post-fertilization; mpf, month post-fertilization.

Because the skin-specific overexpression of human myrAkt1 is not lethal to transgenics, it is possible to observe the late phenotype in Tg(*krt4:Hsa.myrAkt1*)^cy18^. When Tg(*krt4:Hsa.myrAkt1*)^cy18^ reach sexual maturation, the most noticeable phenotypic characteristic is their superior growth rate. We measured the body length and body weight of both genders at one month intervals from 3 to 5 months, and found the body weight but not the body length of both genders display significant increase in Tg(*krt4:Hsa.myrAkt1*)^cy18^ (Table 1). Conditional factor, which compares weight and length, defines the fishes mass index. This resembles human body mass index (BMI), and is a good index for measuring obesity in fish. The Tg(*krt4:Hsa.myrAkt1*)^cy18^ showed a significant increase of conditional factors in both male (37–89% increased, Fig. 4A) and female (84–311% increased, Fig. 4B) compared to WT siblings.

**Table 1:** Metabolic characteristics of WT and Tg(*krt4:Hsa.myrAkt1*)^cy18^ aged at 5 month-old.

Characteristic	WT male	Tg male	WT female	Tg female
Sample size	7	4	14	12
Standard length (cm)	2.86±0.05	2.88±0.09	2.82±0.08	2.90±0.22
Body weight (g)	0.36±0.03	0.41±0.02*	0.46±0.03	0.55±0.12***
Conditional factor, (Kg^3^/m)*101^2^	1709.6±443.4	2350.2±327.1*	3389.6±492.2	6436.2±3900.9***
Total triglycerides content (mg/g)	44.45±17.78	124.71±34.44***	87.76±30.54	252.94±148.65***
Total cholesterol content (mg/g)	25.12±4.64	29.62±13.83	13.82±7.61	22.55±10.00

Stars indicate significant differences as tested by Student’s t-test (*p<0.05, **p<0.01, and ***p<0.001). WT, wild type; Tg, Tg(*krt4:Hsa.myrAkt1*)^cy18^.

In addition to body growth rate, a striking example of phenotypic transformation is the appearance of ectopic bulges in the head, subcutaneous epidermis, and tail fins for founders or stable transgenics carrying *krt4:myrAkt1* transgene at the adult stage (Fig. 4D). The morphology and white color of the bulges suggests that this ectopic structure in Tg(*krt4:Hsa.myrAkt1*)^cy18^ is caused by abnormal fat accumulation. We tested this speculation by Oil Red O staining in the whole animal for WT (Fig. 4C), F0 founder (Fig. 4D), and F1 Tg(*krt4:Hsa.myrAkt1*)^cy18^ (Figs. 4E and 4F). Results reveal heavily red stained lipid areas in the protruding bulges on the face and tail regions of trangenics. When *myrAkt1* transgenes are somatically integrated, F0 founders display ectopic fat accumulation (Fig. 4D). However, when *myrAkt1* transgenes are germ-line integrated, stable transgenics display more evenly distributed fat accumulation over the entire fish at 3 months (Fig. 4E). In 12 month-old fish, the obese Tg(*krt4:Hsa.myrAkt1*)^cy18^ showed more pronounced lipid accumulation along the entire body (especially in the tail fin and visceral regions) and an up-bent body shape (Fig. 4F). The lipids consist primarily of triglycerides and cholesterol. Measurements of the whole body total triglycerides (TG) and total cholesterol (TC) content in Tg(*krt4:Hsa.myrAkt1*)^cy18^ at 5 months showed a 64% to 65% increase in total triglyceride content for male and female Tg(*krt4:Hsa.myrAkt1*)^cy18^, respectively, compared to WT siblings (Table 1). However, the total cholesterol content, show no significant difference between WT and Tg(*krt4:Hsa.myrAkt1*)^cy18^ for both genders (Table 1). These results indicate that the lipid accumulation in obese Tg(*krt4:Hsa.myrAkt1*)^cy18^ primarily consists of excess triglycerides, and not cholesterol accumulation.

**Figure 5:**
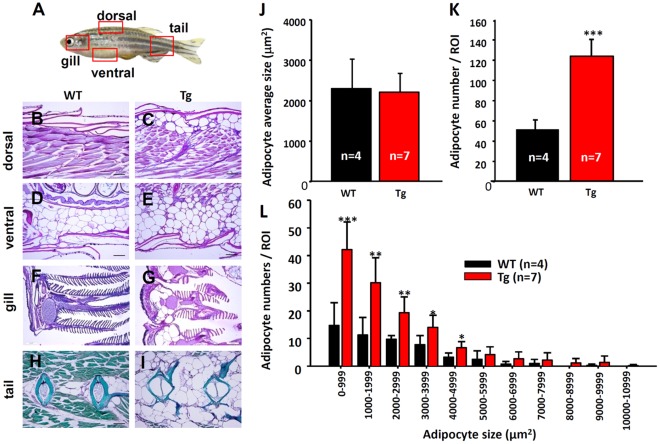
Histology of the obese transformed Tg *(krt4:Hsa.myrAkt1*) ^cy18^ **.** (A) Schematic diagram showing the relative positions (red dotted boxes) for histological sections from B to I. Histological assessment of dorsal muscle tissues (B and C), visceral adipocytes (D and E), gill arch (F and G) and bone tissues in the tail (H and I) for WT and Tg aged 3 mpf. The paraffin sections in B to G were stained with Periodic acid-Schiff, and bone tissues in the tail regions (H&I) were stained with Masson’s trichrome. Comparison adipocyte average size (J), adipocyte numbers (K), and adipocyte size distribution histogram (L) between WT and Tg. Stars above the error bars indicate significant differences as tested by Student’s t-test (*p<0.05, **p<0.01, and ***p<0.001). WT, wild type; Tg, Tg(*krt4:Hsa.myrAkt1*)^cy18^; mpf, month post-fertilization.

Next, we would like to characterize the initiation time and the initiation site for Tg(*krt4:Hsa.myrAkt1*)^cy18^ to display obese phenotype. We performed nile red vital staining on living larvae/juvenile at 7 day intervals to observe the lipid deposition over time. From 7 to 21 dpf, there was no detectable difference in nile red staining patterns between WT and Tg(*krt4:Hsa.myrAkt1*)^cy18^. From 21 dpf onwards, the nile red^+^ adipose tissues located adjacent to and under the posterior swimming bladder showed more pronounced fluorescent staining intensity and cell volume in Tg(*krt4:Hsa.myrAkt1*)^cy18^ (Figs. 4L, 4N, 4P) than their WT siblings (Figs. 4K, 4M, 4O). Therefore, the suitable observation obese transformation in Tg(*krt4:Hsa.myrAkt1*)^cy18^ is detectable at the juvenile to adult transitory stage. Next, we examined the histological observation of adipocyte distribution pattern between WT and transgenics. An entire adult fish aged 5 to 6 months old was sagittally sectioned to determine the distribution of adipose tissues in WT and Tg(*krt4:Hsa.myrAkt1*)^cy18^ (Fig. 5A). In WT, adipose tissues were rarely detected in the compartment between the scale and muscle of the dorsal body (Fig. 5B), but restricted to compartments surrounding the visceral organs (Fig. 5D) and bone (Fig. 5H). However, in obese transformed Tg(*krt4:Hsa.myrAkt1*)^cy18^, some ectopic adipocytes were detected in the compartment between the scale and muscle of the dorsal body (Fig. 5C) and the gill arch (Fig. 5G). In addition, Masson’s trichrome staining revealed bone cells in the vertebrate column and skeletal muscle cells infiltrated and replaced by excess adipocytes in Tg(*krt4:Hsa.myrAkt1*)^cy18^ (Fig. 5I).

The obese transformation and abnormal fat accumulation in Tg(*krt4:Hsa.myrAkt1*)^cy18^ might be caused by adipocyte hypertrophy or hyperplasia. We addressed this question by quantifying adipocyte cell and size in the visceral region. Statistical analysis reveals the adipocyte number (Fig. 5K), but not size (Fig. 5J), in the visceral region was higher in Tg(*krt4:Hsa.myrAkt1*)^cy18^ than in WT. Adipocyte size distribution histogram shows that the obese Tg(*krt4:Hsa.myrAkt1*)^cy18^ is primarily caused by an increase of small adipocytes (Fig. 5L). Collectively, these results confirm that the extreme obese phenotype detected in Tg(*krt4:Hsa.myrAkt1*)^cy18^ is primarily caused by adipocyte hyperplasia rather than hypertrophy.

### The Obese Transformation in Adult Tg(*krt4:Hsa.myrAkt1*)^cy18^ Is Contributed by the Ectopic Expression of myrAkt1

**Figure 6:**
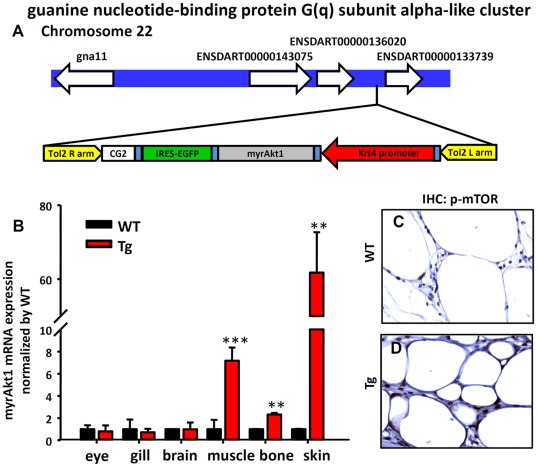
Detection of the ectopic expression of *myrAkt1* transgene in Tg*(krt4:Hsa.myrAkt1*) ^cy18^ **.** (A) Schematic diagram showing the chromosomal integration site in Tg. (B) Relative expression level of *myrAkt1* transgene among different tissues assayed by real-time RT-PCR. Stars above the error bars indicate significant differences as tested by Student’s t-test (**p<0.01, and ***p<0.001). The detection of the expression of a Akt downstream target of phospho-mTOR in the adipose tissues of WT (C) and Tg (D) by immunohistochemistry. WT, wild type; Tg, Tg (*krt4:Hsa.myrAkt1*)^cy18^.

The intriguing obese phenotype in Tg(*krt4:Hsa.myrAkt1*)^cy18^ suggests that using *krt4* promoter to force express human *myrAkt1* gene in the superficial skin layer can boost unexpected obese transformation at the adult stage. Kawakami and colleagues described the random integration nature of *Tol2* transposon system when applied in zebrafish [48], [49]. Therefore, this study hypothesizes that (1) the *krt4:myrAkt1* transgene might integrate into a specific gene to interfere with its expression and function. (2) the *krt4:myrAkt1* transgene might trap neighboring enhancer and misexpress at the adipocyte lineage. This first possibility was tested by exploring the chromosomal integration site in Tg(*krt4:Hsa.myrAkt1*)^cy18^. We extracted genomic DNA, digested it with restriction enzyme, ligated it with adapter, and performed long range PCR to idenify the putative DNA sequences flanking the integration site (Fig. S1). Results show that the *krt4:myrAkt1* transgene was reversely inserted into the intergenic region between two unannotated genes of ENSDART00000136020 and ENSDART00000133739, which were located in a guanine nucleotide-binding protein G(q) subunit alpha-like gene cluster on chromosome 22 (Figs. 6A and S1). Therefore, the obese transformation in Tg(*krt4:Hsa.myrAkt1*)^cy18^ is probably not caused by insertional mutagenesis. Next, we performed real-time RT-PCR to evaluate the relative expression levels of human *myrAkt1* gene in multiple tissues. In addition to its original expression territory in skin (62 fold upregulation), *myrAkt1* transcripts were ectopically detected in liver (3 fold upregulation), muscle (7 fold upregulation), and bone (2 fold upregulation) of Tg(*krt4:Hsa.myrAkt1*)^cy18^ unlike WT siblings (Fig. 6B). Immunohistochemistry on paraffin sections with Akt downstream target antibody also showed stronger p-mTOR-immunoreactive signals in the internal adipose tissues of Tg(*krt4:Hsa.myrAkt1*)^cy18^ (Fig. 6D) than WT (Fig. 6C).

To test whether the ectopic expression of *myrAkt1* transgene is influenced by the enhancer activity of the neighbor genes located adjacent to the transgene integration site, total RNA were extracted from multiple tissues of transgenic fish and performed real-time RT-PCR to compare the relative mRNA expression level of ENSDART00000133739 and ENSDART00000136020 genes. Results showed the relative expression level of ENSDART00000133739 is very low (compared to *β-actin2*) and majorly expressed in the gill tissue (Table S2). The relative abundance of ENSDART00000133739 transcripts is distinct from the *myrAkt1* transcripts detected in Tg(*krt4:Hsa.myrAkt1*)^cy18^. For ENSDART00000136020, it behaviors as a pseudogene since real-time RT-PCR assay failed to detect any gene transcript in either whole fish, whole embryos or adult tissues (Table S2). In addition, no expressed sequence tags (EST) corresponding to ENSDART00000136020 can be detected in Zebrafish EST database. Therefore, the obese transformation in Tg(*krt4:Hsa.myrAkt1*)^cy18^ is probably not contributed by insertional mutagenesis or enhancer trapping of the adjacent neighbor genes. In addition, the obese transformation phenotype is unique to Tg(*krt4:Hsa.myrAkt1*)^cy18^ but not appear in other independent lines carrying the same injecting plasmid. Taken together, these results prove that the obese phenotype in Tg(*krt4:Hsa.myrAkt1*)^cy18^ is caused by the ectopic expression of the *myrAkt1* gene which might be influenced by other long distance enhancer activity.

### Myogenic, Skeletogenic, Adipogenic, Adipocytokine, and Inflammation-related Genes are Deregulated in Tg(*krt4:Hsa.myrAkt1*)^cy18^

**Figure 7:**
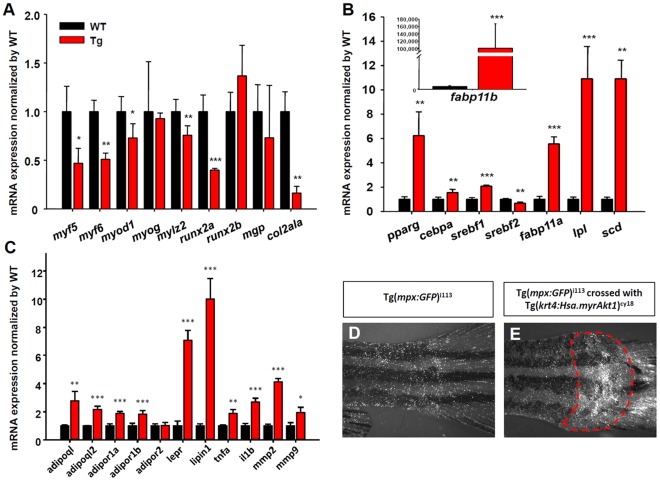
Deregulation of myogenesis-, skeletogenesis-, adipogenesis-, adipocytokine- and inflammation-related genes in Tg*(krt4:Hsa.myrAkt1*)^cy18^. Comparison of relative expression level of myogenesis−/skeletogenesis- (A), adipogenesis- (B), adipocytokine and inflammatory-related (C) gene transcripts between WT and Tg assayed by real-time RT-PCR. Stars labeled above the error bars indicate significant differences as tested by Student’s t-test.*p<0.05, **p<0.01, and ***p<0.001.Visualization of the inflammatory response (red dotted line) in the tail region of both Tg(*mpx:GFP*)^i113^ (D) and double transgenic progeny derived from Tg(*mpx:GFP*)^i113^ and Tg(*krt4:Hsa.myrAkt1*)^cy18^ crossing (E).WT, wild type; Tg, Tg(*krt4:Hsa.myrAkt1*)^cy18^.

The obese transformation of dorsal muscle tissues and tail bone tissues suggest that the normal myogenic and skeletogenic program might be interfered in Tg(*krt4:Hsa.myrAkt1*)^cy18^. To clarify this speculation, we examined the mRNA expression profile of key genes on regulating myogenesis and skeletogenesis using real-time RT-PCR. Myogenic factor 5 (*myf5*), myogenic factor 6 (*myf6*), myogenin (*myog*), myogenic differentiation 1 (*myod1*), and skeletal muscle myosin light polypeptide 2 (*mylz2*) are critical myogenic regulatory factors and structural proteins controlling myogenesis [50], [51], [52], [53], [54], [55]. Results show that most gene transcripts (excluded *myog*) were downregulated in Tg(*krt4:Hsa.myrAkt1*)^cy18^ (Fig. 7A). For skeletogenesis, runt-related transcription factor 2 (*runx2*) is a master osteoblast-specific transcription factor that plays an essential role in osteoblast differentiation and skeletal morphogenesis in mammals [56], [57]. Matrix gla protein (*mgp*) and collagen type II, alpha-1a (*col2a1a*) are key players in the organization of cartilage tissues [58], [59], [60]. Real-time RT-PCR revealed a significant downregulation of *runx2a* and *col2a1a* in Tg(*krt4:Hsa.myrAkt1*)^cy18^ (Fig. 7A). In addition, the genes related to adipogenesis and lipogenesis were strongly upregulated in Tg(*krt4:Hsa.myrAkt1*)^cy18^. The adipogenesis analysis in this study focused on two master transcription factors of peroxisome proliferator-activated receptor gamma (*pparg*) and CCAAT/enhancer binding protein α (*cebpa*) because the interplay between *pparg* and *cebpa* is a crucial in activating adipocyte differentiation and adipogenesis-related program [36], [37], [38]. To test fatty acid transport, triglyceride, cholesterol synthesis, and lipogenesis, we monitored the expression of fatty acid-binding proteins (*fabp11a* and *fabp11b*) [29], sterol regulatory element binding transcription factor 1 (*srebf1*) and *srebf2* [61], lipoprotein lipase (*lpl,* for lipoprotein metabolism) and stearoyl-CoA desaturase (*scd*, for unsaturated fatty acid biosynthesis), respectively. As expected, most gene transcripts (except *srebf2*) showed 2-110,000 fold upregulation in Tg(*krt4:Hsa.myrAkt1*)^cy18^ (Fig. 7B). In summary, the gene expression profile assayed by real-time RT-PCR agreed with the findings collected from morphological and biochemical analysis in the obese transformed Tg(*krt4:Hsa.myrAkt1*)^cy18^.

The global upregulation of gene transcripts in adipogenic and lipogenic program is not surprising because they are the consequences of obesity. To identify unknown factors controlling the obese transformation, we performed oligonucleotide microarray analysis to compare gene transcriptome between WT and Tg(*krt4:Hsa.myrAkt1*)^cy18^ at five months. Compared to WT, 68 and 179 genes were either significantly up or downregulated in Tg(*krt4:Hsa.myrAkt1*)^cy18^. Pathway analysis based on gene ontology identified the inflammatory response pathway (like *mmp2* gene) as a major hub among all upregulated genes (Fig. S2). In mammals, obesity is directly associated with chronic inflammatory response [62]. The adipose tissue in obese animals can interact with neighboring neutrophils or macrophages by releasing adipocytokine or macrophage-derived factors [63]. This microarray data inspired us to test whether the inflammatory response was triggered in the obese transformed zebrafish. We initially validated this hypothesis by examining the expression of adiponectin (*adipoql* and *adipoql2*), adiponectin receptors (*adipor1a*, *adipor1b* and *adipor2*) [64], leptin receptor (*lepr*), and *lipin1*. Systematic analysis shows that the majority of the adipocytokines (except *adipor2*) are upregulated in Tg(*krt4:Hsa.myrAkt1*)^cy18^ (Fig. 7C). Next, we examined the expression level of inflammatory genes released from macrophages. Again, most of the macrophage-derived factors such as *tnfα, il1β, mmp2,* and *mmp9* are strongly upregulated in Tg(*krt4:Hsa.myrAkt1*)^cy18^ (Fig. 7C). To confirm the inflammatory response at cellular level, we crossed Tg(*krt4:Hsa.myrAkt1*)^cy18^ with Tg(*mpx:GFP*)^i113^ to produce a double transgenic line of Tg(*krt4:Hsa.myrAkt1*)^cy18^; Tg(*mpx:GFP*)^i113^ to highlight the green neutrophils in the obese background. We observed the relative number of neutrophils under the fluorescent microcope and found the neutrophils in normal condition were widely and evenly distributed in the whole fish (Fig. 7D). However, the tail region of obese transformed Tg(*krt4:Hsa.myrAkt1*)^cy18^ exhibited extremely strong fluorescent signals because of the unusually high level of neutrophil aggregation (Fig. 7E, highlighted with a red dotted circle). Taken together, these results clearly show that the ectopic activation of human myr*Akt1* in zebrafish mesenchymal derived tissues may activate adipogenesis at the expense of other mesenchymal cells. This ultimately leads to obese transformation in zebrafish.

### Tg(*krt4:Hsa.myrAkt1*)^cy18^ Displays Obese-Related Disease Phenotype

**Figure 8:**
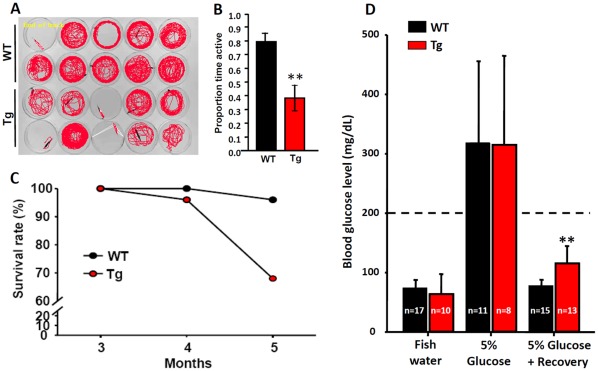
Comparison of swimming activity, survival rate and blood glucose level between wild-type and Tg(*krt4:Hsa.myrAkt1*)^cy18^. (A) Swimming paths of WT and Tg fish in a 5 min window. (B) Active time of swimming in WT and Tg fish. (C) Comparison of the survival rate of WT (black) and Tg (red) at 3 to 5 mpf. (D) The glucose tolerance of WT (black bars) and Tg (red bars). Fish in the 5% glucose positive recovery group were initially immersed in 5% glucose for one day, transferred to fresh fish water for one more day, and finally sacrificed to measure blood glucose level. The dotted line indicates the hyperglycemia level (200 mg/dL). Stars above the error bars indicate significant differences as tested by Student’s t-test.**p<0.01. mpf, month post-fertilization.

The lipoma-like obese transformation in Tg(*krt4:Hsa.myrAkt1*)^cy18^ suggests that the normal physiology has been deregulated and is replaced with a metabolic syndrome-like phenotype. A swimming behavior assay revealed that the obese fish were less active than WT (Figs. 8A and 8B). This reduction of swimming ability might be caused by the loss of muscle, which was replaced by ectopic fat (Fig. 5I). In addition, the survival rate of obese fish was significantly lower than that of WT (Fig. 8C), and most obese were not able to survive longer than two years (data not shown).

To test the blood glucose intolerance, we compared the fasting, feeding, and post-feeding blood glucose levels of adult WT and Tg(*krt4:Hsa.myrAkt1*)^cy18^. This assay is routinely applied in rodents to test whether the experimental animal displays insulin resistance, as indicated by the modulation of blood glucose levels. To model the fasting condition, fish were not fed for 24 hr before blood sampling. To model the feeding condition, fish were initially immersed in 5% glucose solution for 24 h to raise the blood glucose level. In both cases, blood was sampled by decapitation to measure glucose levels. In the fasting condition, the blood glucose level of WT (73±15 mg/dl) and transgenics (64±34 mg/dl) was tightly regulated at less than 100 mg/dl (Fig. 8D). After challenging with 5% glucose for 24 hr, the blood glucose in both WT (317±138 mg/dl) or transgenics (315±150 mg/dl) was greatly elevated, exceeding the critical level of hyperglycaemia (200 mg/dl) [30]. However, compared to WT (77±11 mg/dl), Tg(*krt4:Hsa.myrAkt1*)^cy18^ fish exhibited impaired glucose clearance (115±29 mg/dl) during the recovery period (Fig. 8D). This result indicates that changes in adiposity can reduce glucose tolerance in Tg(*krt4:Hsa.myrAkt1*)^cy18^.

## Discussion

### Akt1 Functions as a Powerful Cell/Organ Size Controller

The over expression of the constitutively active form of human myrAkt1 can phosphorylate endogenous downstream targets like mTOR and 70S6K. This in turn boosts the hyperplastic and hypertrophic growth of zebrafish skin cells. The morpholino-based loss-of-function approach provides direct evidence supporting the skin hyperplasia/hypertrophy in Tg(*krt4:Hsa.myrAkt1*)^cy18^ mediated by Akt1-mTOR-70S6K pathway activation. These findings agree well with previous studies showing that Akt signaling is essential for controlling cell and organ size in mammalian heart [65], [66], [67], skeletal muscle [68], and whole organism [40], [41]. Therefore, these findings confirm that Akt1 plays an evolutionary conserved role in controlling cell and organ size, from fly to mammal. In contrast, careful examination of skin morphology at histological and cellular levels failed to reveal any sign of cancer transformation in Tg(*krt4:Hsa.myrAkt1*)^cy18^ skin. This observation is inconsistent with previous observations in mice because the single activation of myrAkt1 induces spontaneous skin cancer formation [45]. The exact reason for this species-specific difference of Akt1 function on skin is currently unknown, but might be related to the basic physiological difference between endothermic and ectothermic vertebrates. In endothermic vertebrates, only the stem cells located in the basal layer have mitotic potential [69]. In ectothermic vertebrates, cells in all epidermal cell layers are capable of undergoing mitotic division [70]. Recent research demonstrates that skin-specific co-activated Hh signaling with *myrAkt1* in zebrafish skin can induce oncogenic transformation in many tissues, but not in skin itself [71]. In the same manner, this study finds no skin transformation in single transgenic carrying *myrAkt1*. This suggests that fish skin might be more resistant to oncogenic transformation compared to their mammalian counterparts. Future research involving a transgenic zebrafish line carrying multiple oncogenic genes might be able to overcome this oncogenic transformation threshold.

### Tg(*krt4:Hsa.myrAkt1*)^cy18^ Provides a New Lower Vertebrate Obesity Model

Adipocytes originate from meschymal stem cells, and their differentiation is tightly controlled by *pparg* and *cebpa* transcriptional factors. The positive regulatory loop between *pparg* and *cebpa* can activate the terminal differentiation cascade of adipogenesis. Although many in vitro studies have indicated that the *Akt* function is upstream of *pparg* and *cebpa* in modulating adipogenesis [36], [37], [38], [39], [40], [41], the in vivo evidence to support *Akt* function on adipogenesis remains limited. Only a few reports have shown that mice deficient in both *Akt1/Akt2* will display the obese phenotype [40]. This study is the first to use the gain-of-function approach to provide direct evidence that *Akt1* function is required to activate adipogenesis in zebrafish. Using the transgenic approach, Song and Cone generated a zebrafish obese model of Tg(*β-actin:AgRP*) by overexpressing the appetite-enhancing gene of *AgRP* under the control of a ubiquitious *β-actin* promoter [32]. This study develops a new generation of obese fish model of Tg(*krt4:Hsa.myrAkt1*)^cy18^, using *myrAkt1* to directly activate adipogenesis and enhance the hyperplastic growth of adipocytes. Compared to Tg(*β-actin:AgRP*), the Tg(*krt4:Hsa.myrAkt1*)^cy18^ obesity model contains several interesting phenotypes: (1)The over expression of *AgRP* and *myrAkt1* has a significant effect on adipocyte differentiation. Both hypertropic and hyperplastic growth of adipocyte appear in Tg(*β-actin:AgRP*), while only the hyperplastic growth of adipocyte appears in Tg(*krt4:Hsa.myrAkt1*)^cy18^. (2) Tg(*krt4:Hsa.myrAkt1*)^cy18^ exhibits abnormal lipoma-like fat tissue accumulation in dorsal muscle tissues, gill arches, and tail bone tissues, whereas Tg(*β-actin:AgRP*) displays a normal adipocyte distribution. (3) The Tg(*krt4:Hsa.myrAkt1*)^cy18^ displays blood glucose intolerance, but Tg(*β-actin:AgRP*) does not. Therefore, the lipoma-like Tg(*krt4:Hsa.myrAkt1*)^cy18^ reported in this study serves as a good disease model for modeling obesity-induced chronic disease according to the following phenotypic signatures: a great increase in weight gain (Figs. 4A and 4B) and whole body triglyceride content (Table 1), a symptom of muscle contraction weakness (Fig. 8B) and osteoporosis by adipocyte infiltration (Fig. 5J), high neutrophil infiltration (Fig. 6E), glucose intolerance (Fig. 7), and short life span (Fig. 8C). Therefore, Tg(*krt4:Hsa.myrAkt1*)^cy18^ provides a unique and valuable lower vertebrate model to study the mechanism of obesity-induced metabolic deregulation for the first time.

### Lipoma Formation is Associated with the Unbalance of Akt Signaling

Lipoma is a kind of benign, soft tissue tumor characterized by abnormal cell proliferation in adipose tissue. Although cytogenetic research shows that human lipoma formation is associated with the translocations and fusion of HMGA2-NFIB [72], HMGIC-LPP [73], and C11orf95-MKL2 [74], there is little monogenic evidence of lipoma formation. By deleting the PTEN gene in the skeletal lineage, Hsieh and colleagues unexpectedly discovered abnormal lipoma formation in bone and muscle tissues [75]. PTEN is a tumor suppressor gene frequently detected in sporadic human cancers. PTEN negatively regulates the Akt survival signaling by dephosphorylating PIP3 [76]. Once PTEN activity is compromised, the Akt signal is no longer suppressed and the activated Akt can subsequently activate many downstream targets on mediating cell growth, cell survival, and metabolism [77]. The myrAkt1 gain-of-function transgenic fish in this study also display abnormal lipoma transformation in skin, bone, and muscle, agreeing well with the findings reported in PTEN-deficient mice. The lipoma transformation phenotype detected in Tg(*krt4:Hsa.myrAkt1*)^cy18^ is closely related to superficial subcutaneous lipoma (appearing in skin), chondroidlipoma (appearing in bone) and well-differentiated liposarcoma (appearing in muscle), which have been clinically reported in human patients. Therefore, the proposed obese zebrafish model confirms that PTEN-Akt signaling plays an evolutionary conserved role in controlling adipose tissue development. The obese transformed zebrafish model is also a valuable tool for studying obese-related chronic disease at the organism level.

## Materials and Methods

### Animals

The *AB* strain zebrafish (*Danio rerio)* were obtained from ZIRC (http://zebrafish.org/zirc/home/guide.php), and kept in the stock of Chung Yuan Christian University. Zebrafish were raised in local tap water at 28.0±0.5°C under a constant 14 hour light/10 hour dark cycle. After spawning, Zebrafish embryos were collected in 10 cm Petri dishes containing 20 mL fish water and raised at 28.0±0.5°C. To prevent disease from attacking zebrafish embryo, a few drops methylene blue were added to the fish water. At 5 to 7 dpf, larvae were transferred to 10 L tanks containing 8 L of fish water and fed with live Paramecium. After 14 dpf, larvae were fed two times daily with live artemia (OSI, USA) until they reached adulthood.

### Plasmid Construction

We used Tol2 kit [42] to rapidly assemble expression vectors by three-fragment gateway recombination cloning. To create the 5′ entry clone, we amplified 2.2 kb *krt4* promoter from genomic DNA by PCR with forward primer (5′-GGGGACAACTTTGTATAGAAAAGTTGCCTTCCCTTCTACTTTTGACGTCC -3′) and reverse primer (5′-GGGGACTGCTTTTTTGTACAAACTTGCCGGATC-CTGTGTCTTTGAGTTGC-3′). The attB4 and attB1r sites were added at the 5′end of the primers and highlighted by underlines. The PCR products were then cloned into pDONRP4-P1R (Invitrogen) by BP reaction to obtain p5E-krt4. The resulting p5E-krt4 vector contains 2.2 kb upstream regulatory sequences of *krt4* gene that is sufficient to drive target gene specifically express in the superficial skin cells [71]. To create the middle entry clone, we amplified human *myrAkt1*cDNA from plasmid myrAkt delta4-129 (Addgene 10841) using forward primer (5′-GGGGACAAGTTTGTACAAAAAAGCAGGC-TATGGGGAGTAGCAAGAGCAAGC-3′) and reverse primer (5′-GGGGACCACT-TTGTACAAGAAAGCTGGGTTCAGGCCGTGCCGCTGGCCGAG-3′). The attB1 and attB2 sites were added at the 5′end of primers and highlighted by underlines. The PCR products were cloned into pDONR221 (Invitrogen) to generate pME-myrAkt1. The resulting pME-myrAkt1 vector contains the constitutively active form of human Akt1 gene with the 14 aa src myristoylation signal fused to the N terminus of human Akt1 delta 4–129. Finally, p5E-krt4, pME-myrAkt1, and p3E-IRES-EGFPpA [42] were joined with pDestTol2CG2 [42] by LR reaction to create the expression vector of pDestTol2CG2-krt4-myrAkt1-IRES-EGFP-pA.

### Microinjection and Identification of Transgenic Zebrafish

Transposase RNA was synthesized in vitro using pCS-transposase plasmid (kindly provided by Dr. Koichi Kawakami) as a template. DNA was linearized with *Not*I at 37°C overnight and cleaned using DNA Clean/Extraction Kit (GeneMark Inc., Taiwan). Capped mRNA was synthesized by mMESSAGE mMachine SP6 Kit (Ambion). To generate the transgenic zebrafish, we mixed the expression constructs of pDestTol2CG2-krt4-myrAkt1-IRES-EGFP-pA (50 ng/nL) with transposases mRNA (50 ng/nL). Approximately 1–3 nL DNA of a DNA/RNA solution was microinjected into the animal pole of one-cell stage embryos. The injected embryos were raised to adulthood. The putative founders were then identified by the green fluorescent signals in the heart of their F1 progenies, which derived from outcrossing with WT. All experiments were approved by the animal use committee at Chung Yuan Christian University (approval ID. 9815). The transgenic fish line nomenclature of Tg(*krt4:Hsa.myrAkt1*)^cy18^ was approved by the Zebrafish Nomenclature Committee of ZFIN (http://zfin.org).

### Histology

We used a plastic section to analyze the epidermal histology of zebrafish embryo. Zebrafish larvae aged 5 dpf were fixed overnight in 4% paraformaldehyde at 4°C and then dehydrated overnight in 100% methanol at −20°C. After complete dehydration, samples were infiltrated and embedded in Technovit7100 resin (HeraeusKulzer). Samples were sectioned at 1–2 µm intervals and stained with hematoxylin and an eosin staining kit (Merck). To compare the skin thickness and area between WT and transgenic, we took photos at 200× magnification and then selected and processed the region of interesting (ROI) using Photoshop CS3 and Image J software (http://rsbweb.nih.gov/ij/download.html). We used paraffin section to analyze the adipose cell distribution. Adult zebrafish aged 8 months old were first fixed 1 day in 4% PFA and then 3 days in Davidson’s solution (30% ethyl alcohol, 10% Acetic acid, 20% formalin, and 30% ddH_2_O) at room temperature. The samples were then dehydrated with ethanol, cleared with Neo-clear (Merck), and embedded in Paraplast Plus (Leica). Samples were sectioned at 5 µm intervals and stained with Periodic acid-Schiff (PAS) (Merck) or Masson’s trichrome kit (Merck). To compare the adipocyte cell size and number between WT and transgenics, we took photos at 40× magnification and selected and processed a ROI measuring 650 × 650 µm into 8-bit grey scale using Photoshop CS3. We then calculated the cell size and number using Image J software.

### Body Length and Weight Measurement

Mixed gender WT and Tg (*krt4:Hsa.myrAkt1*)^cy18^ embryos were raised in 10 L tanks separately until they reached 3 months old (n = 30 for each group). At this point, WT and Tg(*krt4:Hsa.myrAkt1*)^cy18^ were again sorted according to their gender and subsequently raised in 30 L tanks (n = 7 for males and n = 15 for female). The standard body length and body weight were measured every month until the fish reached five months old, at which point they were sacrificed for lipid measurement.

### Oil Red O Staining

Oil Red O staining was carried out as described by Flynn and colleagues [29]. Zebrafish were fixed in 4% PFA overnight and then washed with PBST for 1 hr at room temperature. After removal from PBST, fish were balanced with 60% isopropanol for 1 hr and immersed in 0.2% Oil Red O in a 60% isopropanol solution overnight and then washed with 60% isopropanol for 1 day.

### Nile Red Staining

Nile red staining was carried out as described by Flynn and colleagues [29]. Zebrafish were incubated in 0.25 µg/ml nile red solution in 0.04% acetone in the dark at 28°C for 30 min and then washed with fish water for 1 min to reduce staining background. After anesthetizing with MS222, fish were mounted in 3% methyl cellulose and imaged using a fluorescence dissecting microscope (Nikon SMZ 1500) equipped with a GFP long-pass filter.

### Measurement of Total Triglyceride and Cholesterol Contents

Total triglyceride and cholesterol in the whole zebrafish was measured using a commercial assay kit (Diasys Diagnostic Systems) and detected by a Synergy HT Multi-Mode Microplate Reader (BioTek Instruments Inc., Vermont, USA). Both wild type and F1 Tg(*krt4:Hsa.myrAkt1*)^cy18^ fish aged 5 months old were sacrificed and completely homogenized into powder in liquid nitrogen. The fish powder was mixed with 5 ml chloroform/methanol (2∶1) to extract lipids, and the soluble fraction was filtered into a 15 ml centrifuge tube. After removing a 10µl sample to combine with 10 µl Triton X-100, the excess solvent was evaporated by a vacuum pump at room temperature and weighed to measure total lipid mass per fish. Mass measurements are reported as mean ± SD.

### Immunostaining and Fluorescence Microscopy

Zebrafish larvae aged 3 dpf were fixed in 4% paraformaldehyde for 1 h at 4°C, followed by 1× PBST washing (PBS with 0.1% Triton X-100) for 30 min at 4°C. Larvae were then dehydrated with 100% methanol for at least 1 h and then kept at −20°C. After PBST washing for 30 min at 4°C, larvae were blocked in 3% BSA/PBST for 1 h at 4°C. Rabbit polyclonal antibody against a short amino acid sequence containing human Akt1 (pan-Akt1/2/3, sc-8312, Santa Cruz) was diluted to 1∶100 in 3% BSA/PBST and incubated with larvae overnight at 4°C. The next day, the larvae were washed 1× PBST for 30 min at 4°C and incubated with a goat anti-rabbit Alexa 488 (Invitrogen) solution diluted to 1∶200 in 3% BSA/PBST for 1 h at room temperature. Images were acquired by a fluorescence dissecting microscope (Nikon SMZ 1500) equipped with an Evolution VF monochrome CCD (Media Cybernetics).

### Western Blot

Tail fins were collected from 20 individuals of either wild-type or Tg(*krt4:Hsa.myrAkt1*)^cy18^ and homogenized in a protein lysis buffer (250 mM sucrose, 20 mM Hepes, 1 mM EDTA, pH7.4, 1% protease inhibitor, Sigma). The lysates were centrifuged at 13,000 rpm for 20 min at 4°C to remove debris, and the supernatant was collected for further analysis. The level of protein concentration was determined by BCA protein assay kit (Thermo) and detected by Synergy HT Multi-Mode Microplate Reader (BioTek Instruments Inc., Vermont, USA). Thirty-five µg of proteins were separated by 12.5% SDS-PAGE and transferred to polyvinylidene difluoride (PVDF) membranes (Millipore). Following incubation with a blocking solution, PVDF membranes were incubated with primary antibodies (1∶1000 dilution) overnight and then incubated with HRP-conjugated secondary antibodies (1∶3500 dilution) for 1 hr at room temperature. The primary antibodies used were listed as follows: rabbit anti-human Akt1/2/3 (sc-8312, Santa Cruz), rabbit anti-human phospho-Akt1/2/3 at Ser 473 position (sc-7985-R, Santa Cruz), rabbit anti-human phospho-GSK3α/β (pY279/pY216) (2309-1, Epitomics), rabbit anti-human p70 S6 kinase alpha (pT421/pS424) (1135-1, Epitomics), rabbit anti-human phospho-mTOR (pS2448) (2971, Epitomics), and mouse anti β-actin (sc-69879, Santa Cruz). After a series of washing, protein bands were detected by incubating in WEST-ZOL PLUS solution (iNtRON Biotechnology, Korea) for 2 min at room temperature in a dark room. Images were acquired using a FUJIFILM LAS 3000 Imaging Analyzer (FUJIFILM, Taiwan).

### Real-time RT-PCR

We dissected two tail tissues (as a pooled sample) taken from six month-old WT and Tg(*krt4:Hsa.myrAkt1*)^cy18^ (n = 5) and homogenized in Trizol reagent (Ambion). The total RNA was isolated following the manufacturer’s instructions. The RNA was treated with DNase I at 25°C for 10 minutes to remove DNA contamination and then cleaned using RNase-free spin columns (Qiagen). The total RNA concentration was determined by spectrophotometry (ND-1000; NanoDropTechnol, Wilmington, DE), and the RNA quality was checked by running electrophoresis in RNA-denatured gels. The total RNA thus extracted was stored at −20°C. For real-time RT-PCR, 1 µg sample of total RNA was reverse-transcribed with reverse transcriptase and then digested with *E.coli* RNase H to enhance the cDNA purity. Real-time RT-PCR was performed in a Roche LightCycler480 following the manufacturer’s instructions. The primer sequence used to perform real-time RT-PCR and their corresponding amplicon size are listed in Table S1.

### Microarray Analysis

The Zebrafish 14 K oligo microarray chip was obtained from the Institute of Cellular and Organismic Biology at Academia Sinica. This chip contained 14,067 oligonucleotides representing 9,666 unique genes with a redundancy of 31%. The detail oligonucleotide description is available from OcimunBiosolutions (http://www.ocimumbio.com/web/default.asp). We used a SuperScript™ Indirect cDNA Labeling System kit (Invitrogen) to generate fluorescently labeled probes. The total RNA isolated from WT and Tg(*krt4:Hsa.myrAkt1*)^cy18^ was reversed transcribed into cDNA and coupled for Alexa Fluor 555 and Alexa Fluor 647 fluorescent dyes (Invitrogen), respectively. Before hybridization, the Zebrafish microarray chips were pretreated with 1% bovine serum albumin, 4× SCC, and 1% sodium dodecylsulfate (SDS) for 45 min at 42°C, and then hybridized in SlideHyb™ buffer (Ambion) overnight at 42°C. After hybridization, chips were washed with 2× SSC and 0.5% SDS for 15 min at 25°C and then again with 0.5× SSC and 0.5% SDS for 15 min at 25°C. The fluorescence intensities of Alexa Fluor 555 and Alexa Fluor 647 targets were determined using a Genepix scanner (Molecular Devices, Sunnyvale, CA, USA) and the acquired data were analyzed using Genepix and Genespring software (Aglient Technologies, Foster City, CA, USA). The microarray data were submitted to NCBI Gene Expression Omnibus (http://www.ncbi.nlm.nih.gov/geo/) under accession numbers GSM542371 to GSM542373.

### Morpholino Oligo Injection

To achieve the maximal knock-down effect, 1 nl of serially-diluted MOs (purchased from Gene Tools) at concentrations of 1, 0.5, 0.25, and 0.1 mM were injected into yolks at the 1-cell stage. The optimal dosage for gene knockdown was as follows: mTOR splicing block MO, 5′-GGTTTGACACATTACCCTGAGCATG-3′ at 5 ng/embryo; 70S6K splicing block MO, 5′-CAGTCTTCAACTTACGTGAACAAGA-3′ at 5 ng/embryo. Previous research has reported the specificity and efficacy of both MOs [78].

### Swimming Behavior Assay

Zebrafish aged 8 months old were individually incubated in 100 mL of fish water in home-made plastic dishes with a 7 cm diameter. Eight samples were collected from either WT or transgenic groups, and their swimming behavior was recorded with a digital camera for 5 min. Each animal’s level of activity was analyzed using video-based animal movement tracking software (EthoVision XT, Noldus).

### Elucidation of Genomic Integration Site

Genomic DNA was extracted from tail fin tissues of Tg(*krt4:Hsa.myrAkt1*)^cy18^ using a commercial kit (GeneMark, Taiwan) and digested with *Stu*I restriction enzyme. The *Stu*I-digested genomic DNA fragments were ligated with an adapter and then subjected to PCR following the manufacturer’s instructions (Genome Walker kit, Clontech). The amplified PCR products were then directly subjected to DNA sequencing and the readouts were performed BLAT search against zebrafish genome database (Zv9 dataset/danRer7) at the UCSC genome browser (http://genome.ucsc.edu/).

### Blood Glucose Measurement

Zebrafish were sacrificed by cutting off the tail and the blood samples were collected with a glass capillary measuring 1 mm in diameter. Blood samples of approximately 5 uL were then applied to the sensor chip of a commercial blood glucose meter (Accu-Chek performa, Roche Diagnostics, detection range  = 10−600 mg/dl) to measure the blood glucose level at fasting, feeding, or after feeding status.

## Supporting Information

Figure S1 **Elucidation of DNA sequences flanking the chromosomal integration site in Tg(** ***krt4:Hsa.myrAkt1*** **)^cy18^.** The genomic DNA sequences flanking the integration site elucidated by linker-mediated PCR are highlighted by blue colors. The Tol2 transposable element sequences flanking the integration site are highlighted by black colors. The footprints of Tol2 integration site are labeled by underlining.
(TIF)

Figure S2 **Network of the deregulated genes in obsese transformed zebrafish.** Red and green text donate genes with increased and decreased expression, respectively, in Tg(*krt4:Hsa.myrAkt1*)^cy18^ when the wild-types are compared. The microarray data of triplicated assay were submitted to NCBI Gene Expression Omnibus under accession numbers GSM542371 to GSM542373.
(TIF)

Table S1 **The PCR amplicon size and primer sequences used to perform real-time RT-PCR.**
(XLS)

Table S2 **Measurement of mRNA expression level of two genes located adjacent to the chromosomal insertion site in multiple tissues by real-time RT-PCR.**
(XLS)
