# Supplementary figures and images for: Overexpression of Akt1 Enhances Adipogenesis and Leads to Lipoma Formation in Zebrafish
Source: PLoS One. 2012 May 18;7(5):e36474. doi: 10.1371/journal.pone.0036474 (PMC3356305; doi:10.1371/journal.pone.0036474)

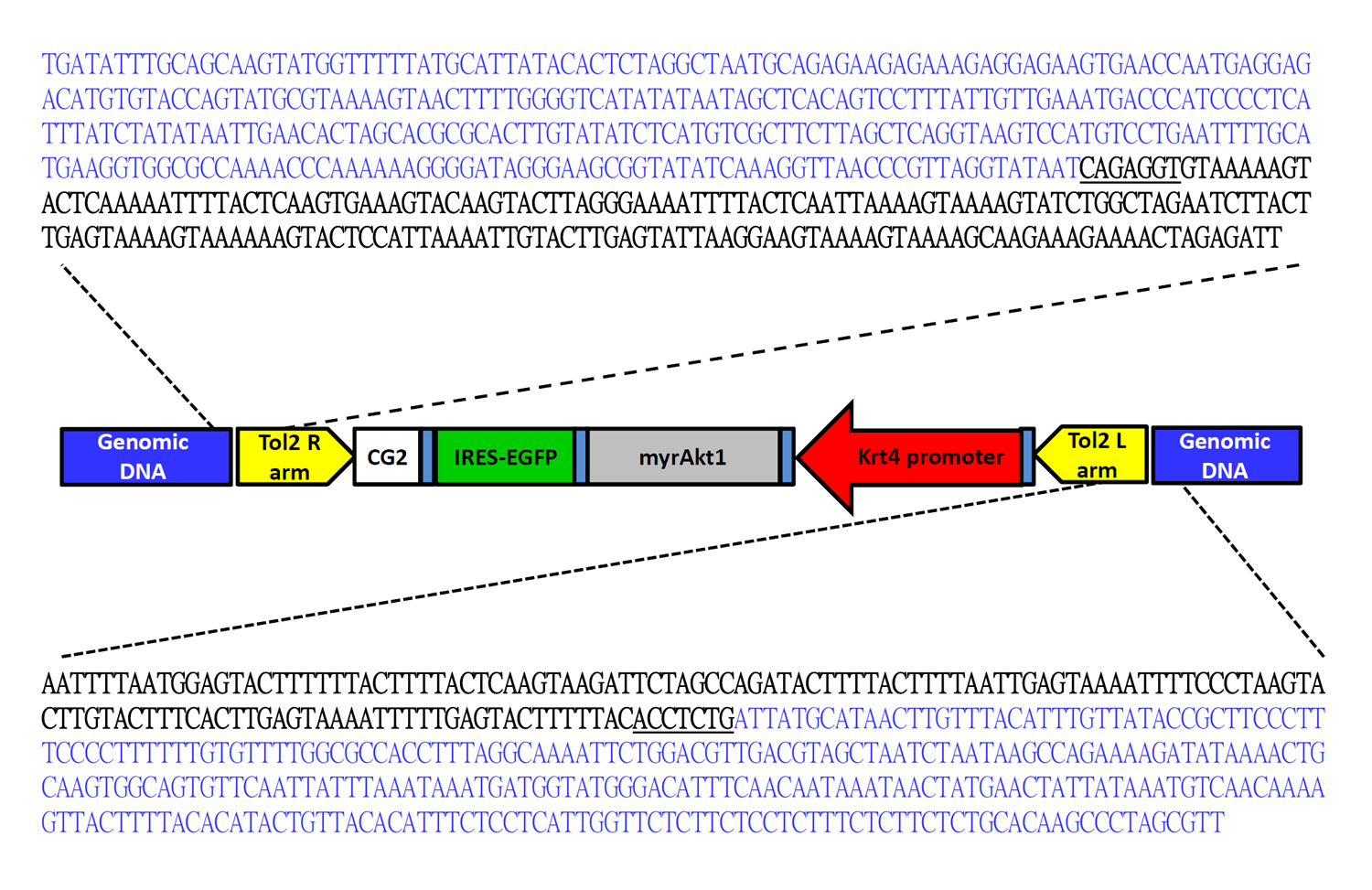

Supplement: Figure S1 — Elucidation of DNA sequences flanking the chromosomal integration site in Tg( krt4:Hsa.myrAkt1 )cy18. The genomic DNA sequences flanking the integration site elucidated by linker-mediated PCR are highlighted by blue colors. The Tol2 transposable element sequences flanking the integration site are highlighted by black colors. The footprints of Tol2 integration site are labeled by underlining. (TIF) [file pone.0036474.s001.tif]

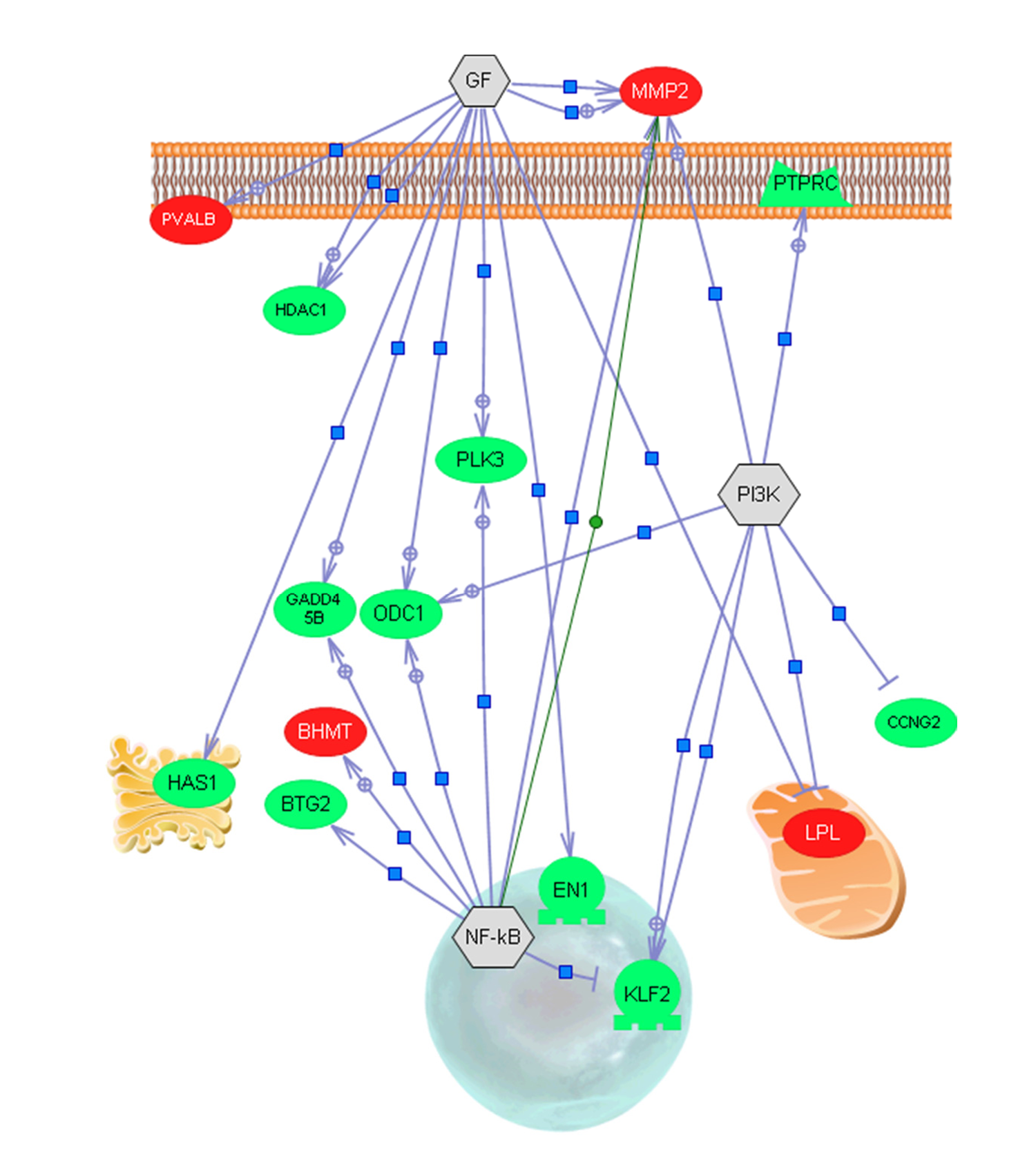

Supplement: Figure S2 — Network of the deregulated genes in obsese transformed zebrafish. Red and green text donate genes with increased and decreased expression, respectively, in Tg(krt4:Hsa.myrAkt1)cy18 when the wild-types are compared. The microarray data of triplicated assay were submitted to NCBI Gene Expression Omnibus under accession numbers GSM542371 to GSM542373. (TIF) [file pone.0036474.s002.tif]
